# *Halothiobacillus neapolitanus* Carboxysomes Sequester Heterologous and Chimeric RubisCO Species

**DOI:** 10.1371/journal.pone.0003570

**Published:** 2008-10-30

**Authors:** Balaraj B. Menon, Zhicheng Dou, Sabine Heinhorst, Jessup M. Shively, Gordon C. Cannon

**Affiliations:** 1 Department of Chemistry and Biochemistry, The University of Southern Mississippi, Hattiesburg, Mississippi, United States of America; 2 Department of Genetics and Biochemistry, Clemson University, Clemson, South Carolina, United States of America; Newcastle University, United Kingdom

## Abstract

**Background:**

The carboxysome is a bacterial microcompartment that consists of a polyhedral protein shell filled with ***r***ib***u***lose 1,5-***bis***phosphate ***c***arboxylase/***o***xygenase (RubisCO), the enzyme that catalyzes the first step of CO_2_ fixation via the Calvin-Benson-Bassham cycle.

**Methodology/Principal Findings:**

To analyze the role of RubisCO in carboxysome biogenesis *in vivo* we have created a series of *Halothiobacillus neapolitanus* RubisCO mutants. We identified the large subunit of the enzyme as an important determinant for its sequestration into α-carboxysomes and found that the carboxysomes of *H. neapolitanus* readily incorporate chimeric and heterologous RubisCO species. Intriguingly, a mutant lacking carboxysomal RubisCO assembles empty carboxysome shells of apparently normal shape and composition.

**Conclusions/Significance:**

These results indicate that carboxysome shell architecture is not determined by the enzyme they normally sequester. Our study provides, for the first time, clear evidence that carboxysome contents can be manipulated and suggests future nanotechnological applications that are based upon engineered protein microcompartments.

## Introduction

**Figure 1:**
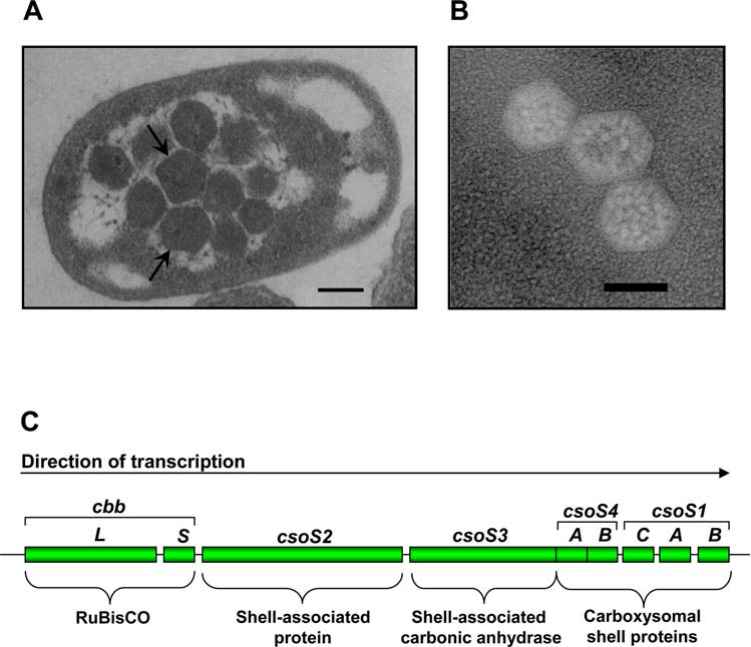
Transmission electron micrographs of carboxysomes. (A) Thin section of a wild type *H. neapolitanus* cell harboring multiple carboxysomes (arrows). (B) Negatively stained purified carboxysomes. (C) The *H. neapolitanus cso* operon, which contains the genes for Form I RubisCO (*cbbL*, *cbbS*) and the carboxysomal shell proteins (*csoS2, csoS3, csoS4A, csoS4B, csoS1C, csoS1A, csoS1B*).

Bacteria, like eukaryotes, contain subcellular structures that function to compartmentalize certain metabolic steps or reaction sequences (reviewed in [1]). By creating a unique environment, these organelles facilitate the chemistry of reactions and/or contribute to the regulation of pathways. While eukaryotic organelles are defined by a lipid bilayer boundary, their prokaryotic counterparts are much simpler structurally, and most of them are not enclosed by a classical biological membrane. The prototype bacterial organelle is the carboxysome (Figure 1), a polyhedral microcompartment found in cyanobacteria and in many chemoautotrophs (reviewed in [2]). The carboxysome consists of a thin protein shell that surrounds a core composed of the CO_2_ fixing enzyme ribulose 1,5-bisphosphate carboxylase/oxygenase (RubisCO, EC 4.1.1.39). Phylogenetically and on the basis of their shell protein complement the α-carboxysomes of chemoautotrophs (incl. *H. neapolitanus*) and many marine cyanobacteria can be distinguished from the β-carboxysomes found mostly in freshwater cyanobacteria [2]. Tightly associated with the shell of α-carboxysomes is a unique carbonic anhydrase that enhances the catalytic efficiency of the sequestered RubisCO by dehydrating abundant cytosolic bicarbonate and providing RubisCO with its substrate, CO_2_. The identity of the carbonic anhydrase of β-carboxysomes (CcmM or CcaA) and its location within the microcompartment are not known and await the purification of β-carboxysomes to homogeneity for analysis of their protein constituents [2].

Another key to the function of the carboxysome is its protein shell. The arrangement of the major structural proteins into tightly packed hexamers with small central pores [3]-[5] creates a boundary that effectively impedes diffusion of CO_2_ out of the carboxysome [6], [7]. The resulting localized high concentration of the RubisCO substrate in the microcompartment interior enhances CO_2_ fixation by the catalytically rather inefficient RubisCO. Whether the carboxysome shell also protects RubisCO from its competing substrate, oxygen, remains to be resolved. Likewise, the molecular mechanisms by which ribulose 1,5-bisphosphate gains entry into the carboxysome interior and by which the two molecules of 3-phosphoglycerate that are the products of the carboxylation reaction are released from the microcompartment are not known.

The importance of carboxysomes for autotrophic metabolism is well documented (reviewed in [2]). Perturbation of genes encoding carboxysomal proteins yields mutants with a ***h***igh ***C***O_2_-***r***equiring (*hcr*) phenotype that grow appreciably only if the atmosphere is supplemented with CO_2_ [6], [8]–[12]. Microcompartmentalization of RubisCO with a carbonic anhydrase thus allows those autotrophic bacteria that form carboxysomes to grow efficiently at ambient CO_2_ levels.

The carboxysomal RubisCO of *H. neapolitanus* and other autotrophs is composed of eight large (CbbL or RbcL) and eight small (CbbS or RbcS) subunits (L_8_S_8_) and is classified as a Form I enzyme [13], [14]. The phylogenetically distinguishable RubisCO types that are sequestered into α- and β-carboxysomes have been assigned to the subclasses IA and IB, respectively [14]. Form IB genes are part of the gene clusters encoding the β-carboxysome only in some cyanobacteria. The genes of the carboxysomal Form IA RubisCO, on the other hand, are always part of the *cso* operon, where they are followed by the genes for the α-carboxysomal shell proteins (Figure 1) [15], [16]. Many chemoautotrophs carry genes for one or two additional RubisCO species (reviewed in [14]). The γ-proteobacteria *Thiomicrospira crunogena*, *Hydrogenovibrio marinus* and *Acidithiobacillus ferrooxidans* carry a second set of genes for a Form I RubisCO species that are not part of their respective *cso* operon [17]–[19]. Several chemoautotrophs also harbor a gene (*cbbM*) for a Form II RubisCO (reviewed in [14]). The Form II RubisCO of *H. neapolitanus* consists of a dimer of large subunits (L_2_). The physiological significance of duplicate RubisCO species in these bacteria is not well understood, but it is known that their respective expression profiles in *H. marinus* respond to inorganic carbon availability [18].

To address the role of its cargo protein in α-carboxysome biogenesis and shell assembly we deleted the genes for the carboxysomal RubisCO from the genome of *Halothiobacillus neapolitanus* and created additional mutants in which the *cbbL* and/or *cbbS* genes were replaced with those from another bacterium. We have characterized the growth phenotypes and the polyhedral microcompartment-like structures formed in these mutants and found that carboxysome shell formation is independent of RubisCO sequestration. We show for the first time that a foreign RubisCO species can be sequestered into carboxysomes. Our results provide the basis for further genetic approaches to elucidate carboxysome biogenesis and assembly and pave the way for their future development for nanotechnological applications.

## Results

### Growth phenotype of *H. neapolitanus* Form I RubisCO replacement mutants

**Figure 2:**
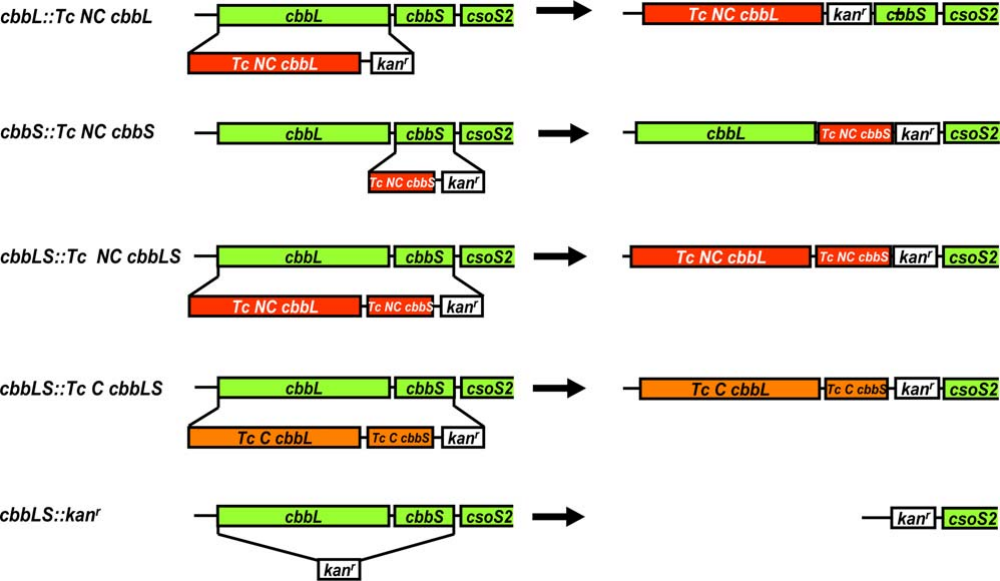
*H. neapolitanus* RubisCO replacement and deletion mutants. The mutants were constructed by replacing the *H. neapolitanus* genes for large (*cbbL*) and small (*cbbS*) subunit (green boxes) with noncarboxysomal (*Tc NC*; red boxes) or carboxysomal (*Tc C*; orange boxes) genes from *T. crunogena* or replacing both genes with a kanamycin resistance cassette (*kan^r^*; white boxes). All mutants carry a kanamycin cassette for selection purposes.

To assess whether the presence of endogenous RubisCO is a prerequisite for α-carboxysome formation, we created a series of *H. neapolitanus* mutants in which the *cbbL* and/or *cbbS* genes that are part of the *cso* operon (Figure 2) were either replaced by orthologs from the γ-proteobacterium *T. crunogena* or deleted altogether. In the *cbbS::Tc NC cbbS* and *cbbL::Tc NC cbbL* mutants, the *T. crunogena* noncarboxysomal (NC) *cbbS* or *cbbL* gene takes the place of the respective endogenous carboxysomal (C) ortholog. These mutants were designed to express chimeric RubisCO molecules. The *cbbLS::Tc C cbbLS* and *cbbLS::Tc NC cbbLS* mutants, in which the genes for both subunits of the *T. crunogena* carboxysomal and noncarboxysomal RubisCO, respectively, substitute for the two RubisCO genes from *H. neapolitanus*, were designed to express heterologous enzyme. Both RubisCO genes were replaced by a kanamycin resistance cassette in the *cbbLS::kan^r^* Form I RubisCO deletion mutant.

**Figure 3:**
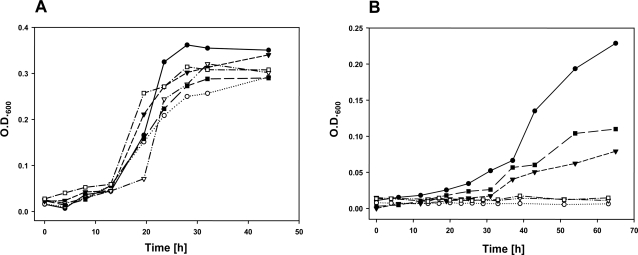
Growth of wild type and RubisCO mutants. Growth of *H. neapolitanus* cultures in air supplemented with 5% CO_2_ (A) and in ambient CO_2_ (B); *wild type* (•), *cbbL::Tc NC cbbL* (○), *cbbS::Tc NC cbbS* (▾), *cbbLS::Tc NC cbbLS* (∇), *cbbLS::Tc C cbbLS* (▪), and *cbbLS::kan^r^* (□). Growth was monitored by measuring optical density of batch cultures at 600 nm.

All RubisCO mutants were able to grow at rates and to maximum densities similar to wild type *H. neapolitanus* in air that is enriched with 5% CO_2_ (Figure 3A). At ambient CO_2_ levels, the *cbbS::Tc NC cbbS* and *cbbLS::Tc C cbbLS* mutants grew considerably more slowly than the wild type, and the *cbbL::Tc NC cbbL*, *cbbLS::Tc NC cbbLS*, and *cbbLS::kan^r^* mutants did not to grow at all over a time period of more than 60 hours (Figure 3B).

### All *H. neapolitanus* replacement mutants express Form I RubisCO

**Figure 4:**
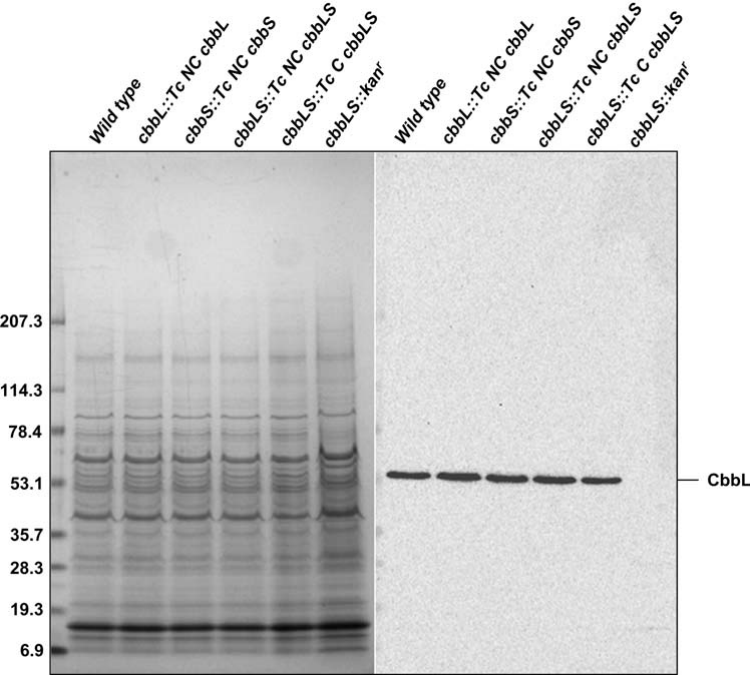
Expression of CbbL in wild type and RubisCO mutants. Clarified cell extracts (10 µg protein) were resolved by SDS-PAGE (left). A blot of an identical gel was probed with an anti-CbbL antibody that is specific for the large subunit of Form I RubisCO species.

The *cbbLS::kan^r^* mutant was not expected to grow in air because this mutant, like the *cbbL* insertion mutant constructed by Baker *et al.* [10], did not produce the carboxysomal Form I RubisCO (Figure 4) and relied instead on utilization of the available intracellular inorganic carbon pool by its Form II RubisCO. The basis for the *hcr* phenotype of the *cbbL::Tc NC cbbL* and *cbbLS::Tc NC cbbLS* mutants was less clear because all replacement mutants were constructed so that the respective *cbbL* and *cbbS* genes remained under the control of the endogenous *H. neapolitanus cso* promoter or of the *kan^R^* promoter (Figure 2) and should therefore produce endogenous and heterologous CbbL and CbbS polypeptides at appreciable levels [20].

To assess RubisCO protein expression in the *H. neapolitanus* mutants, we probed crude extracts of cells that were grown under elevated CO_2_ for the presence of CbbL protein, using a commercially available antibody that is specific for the conserved large subunit of Form I enzymes. All mutants other than *cbbLS::kan^r^* contained near wild-type levels of the respective CbbL protein (Figure 4), indicating that the growth defect of the *cbbL::Tc NC cbbL* and *cbbLS::Tc NC cbbLS* mutants could not be explained by a failure to express the foreign CbbL.

**Figure 5:**
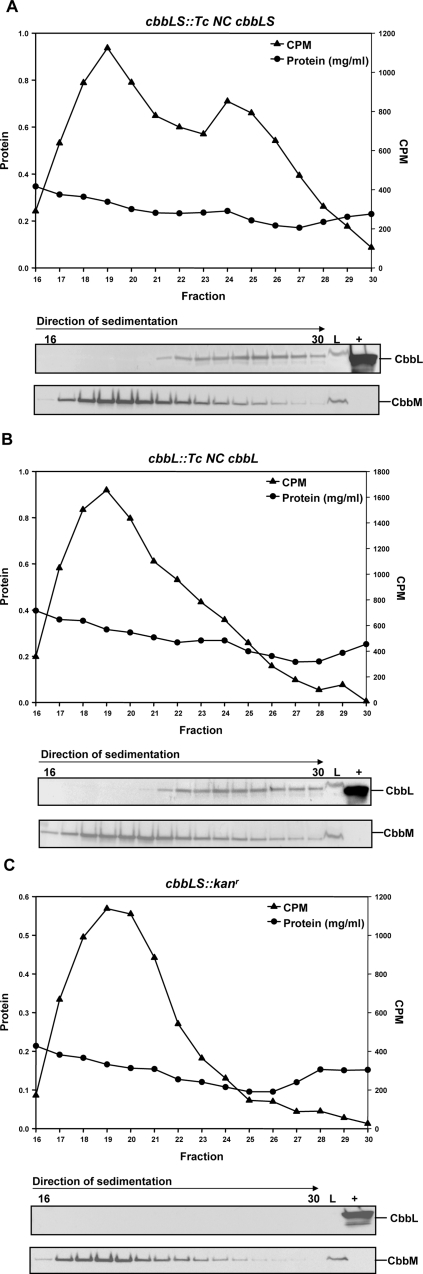
RubisCO activity in cell extracts of the *cbbL::Tc NC cbbL*, *cbbLS::Tc NC cbbLS,* and *cbbLS::kan^r^* mutants. Clarified extracts of *H. neapolitanus cbbL::Tc NC cbbL* (A), *cbbLS::Tc NC cbbLS* (B), and *cbbLS::kan^r^* (C) mutant cells were separated on 0.2–0.8 M sucrose gradients. The resulting fractions were assayed for RubisCO activity (cpm) and protein content (mg/ml). Aliquots (25 µl) of fractions 16–30 were probed for the presence of CbbL and CbbM with antibodies specific for each RubisCO type. L = 5 µg of clarified cell extract prior to gradient centrifugation; (+) = wild type carboxysome control.

To ascertain whether those *H. neapolitanus* mutants that did not grow in air formed enzymatically active RubisCO holoenzyme, we determined RubisCO activity and performed immunoblot analysis after fractionation of cell-free extracts on sucrose density gradients (Figure 5). Under the conditions employed, the large L_8_S_8_ Form I RubisCO molecules sediment far into the gradient, and their activity profile is clearly distinguishable from that of the smaller Form II enzyme [10], which in *H. neapolitanus* consists of a dimer of CbbM subunits. The *cbbLS::Tc NC cbbLS* mutant, in which the *H. neapolitanus cbbL* and *cbbS* genes were replaced with the noncarboxysomal orthologs from *T. crunogena*, contained active heterologous RubisCO holoenzyme, as indicated by a peak of activity and matching immunoblot signals centering around fractions 24–26. The activity of the smaller CbbM enzyme peaked in fractions 19 and 20 (Figure 5A). The Form I RubisCO activity in the *cbbL::Tc NC cbbL* mutant was of similar magnitude as that in *cbbLS::Tc NC cbbLS*, but the peak was less distinct because it was partially masked by the higher CbbM activity in this mutant (Figure 5B). The level of Form I RubisCO activity in these two mutants represented 20–25% of that found in wild type extracts fractionated in a similar fashion. By contrast, the *cbbLS::kan^r^* mutant, as expected, did not express any L_8_S_8_ RubisCO species (Figure 5C).

### All RubisCO mutants produce carboxysome shells

Since in all mutants the genes of the *cso* operon that encode carboxysome shell components either remained under the control of the endogenous *cso* operon promoter or, more likely, were controlled by the promoter of the kanamycin cassette that was inserted between the *cbbS* and *csoS2* genes for selection purposes (Figure 2), these genes were expected to be expressed. We evaluated the ability of the mutants to assemble carboxysome shells and to sequester the mutant RubisCO proteins they produce into the microcompartments. Of particular interest in this regard were the mutants that did not grow at all in air and displayed the most severe *hcr* phenotype, similar to the *hcr* “cyanorubrum” mutant of *Synechocystis* 6803, which only expresses the heterologous Form II RubisCO and no endogenous Form I enzyme and was reported to lack recognizable carboxysomes [21], [22]). The *hcr H. neapolitanus* mutant *cbbL::*Km also does not express Form I RubisCO. This mutant, however, was reported to contain structures that resemble carboxysome shells but are smaller than wild type carboxysomes [10].

**Figure 6:**
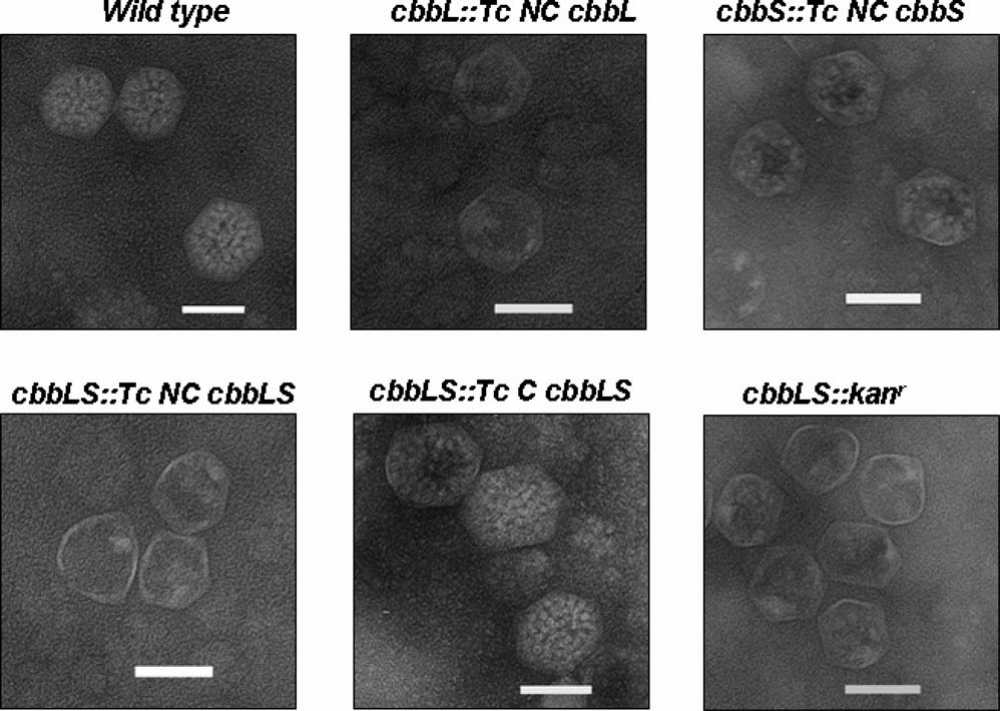
Electron micrographs of wild type and RubisCO mutant carboxysomes. Purified carboxysomes were stained with 1% ammonium molybdate and visualized by electron microscopy at 50,000 X magnification. Scale bars = 100 nm.

We adopted the well-established cell fractionation protocol that is routinely used in our laboratory to purify wild type and mutant carboxysomes [6], [23], [24]. For all mutants employed in this study, the final differential centrifugation step (48,000×*g*) in the enrichment protocol produced the typical pellets that in wild type *H. neapolitanus* are substantially enriched in carboxysomes. Further purification of this fraction by sucrose density centrifugation yielded opaque bands in the gradient at positions similar to those of wild type carboxysomes. Electron microscopy revealed that the bands obtained from all mutants contained polyhedral structures of approximately 100 nm diameter (Figure 6). The polyhedra isolated from the *cbbS::Tc NC cbbS* and *cbbLS::Tc C cbbLS* mutants were filled with RubisCO holoenzyme molecules that in the *cbbLS::Tc C cbbLS* mutant represented heterologous *T. crunogena* carboxysomal RubisCO. In the *cbbS::Tc NC cbbS* mutant, the sequestered chimeric RubisCO species was composed of endogenous *H. neapolitanus* CbbL and noncarboxysomal CbbS from *T. crunogena*. For the *cbbL::Tc NC cbbL*, *cbbLS::Tc NC cbbLS* and *cbbLS::kan^r^* mutants, the polyhedral structures represented apparently intact carboxysome shells that were devoid of RubisCO (Figure 6). To substantiate these observations, mutant carboxysomes were subjected to SDS-PAGE and immunoblotting.

**Figure 7:**
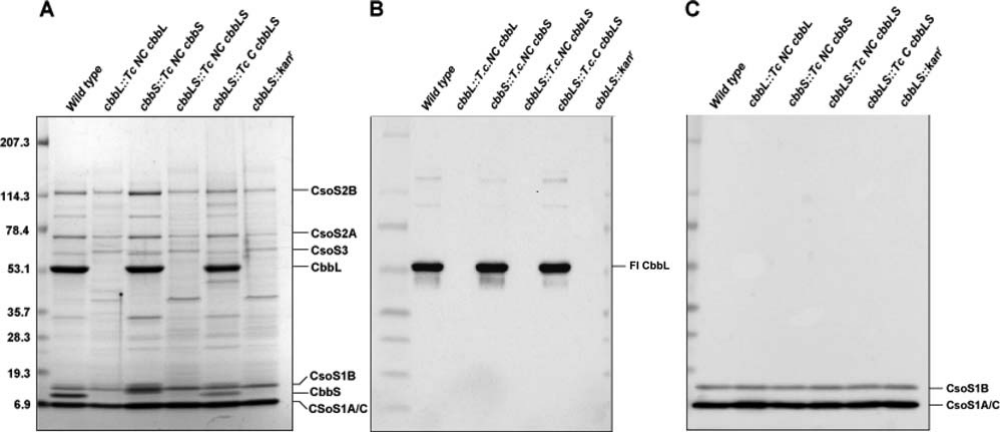
Polypeptide composition of purified carboxysomes. Carboxysome proteins were separated by SDS-PAGE and stained with Gelcode Blue (A). Blots of identical gels were probed with antibodies specific for the large subunit of Form I RubisCO (B) and the CsoS1 shell proteins (C). An equal number of carboxysomes was loaded in each lane.

According to the patterns of stained polypeptide bands (Figure 7A), all empty and filled polyhedral shells were composed of the typical set of carboxysome shell proteins in near wild type stoichiometric ratios. Immunoblots probed with antibodies that recognize all three major carboxysome shell proteins (CsoS1A, CsoS1B and CsoS1C) clearly showed that the CsoS1 proteins were present in all mutant carboxysomes and empty shells at approximately the same levels as in the wild type (Figure 7A,C).

Carboxysomes from the *cbbS::Tc NC cbbS* and *cbbLS::Tc C cbbLS* mutants encapsulated RubisCO like their wild type counterparts (Figure 7A,B). The CbbS band derived from the *cbbS::Tc NC cbbS* mutant particles was located above those of wild type *H. neapolitanus* CbbS and of *T. crunogena* carboxysomal CbbS in the SDS-polyacrylamide gel (Figure 7A). This difference in migration rate reflects the higher molecular weight of the *T. crunogena* noncarboxysomal RubisCO small subunit compared to the carboxysomal CbbS from both bacteria. The presence of *T. crunogena* CbbS and CbbL polypeptides in mutant carboxysome was verified by MALDI-ToF mass spectrometry (data not shown) and indicated that a foreign RubisCO, *T. crunogena* carboxysomal holoenzyme (*cbbLS::Tc C cbbLS*), and chimeric molecules consisting of *H. neapolitanus* CbbL and *T. crunogena* noncarboxysomal CbbS (*cbbS::Tc NC cbbS*) could be incorporated into *H. neapolitanus* carboxysomes. By contrast, the purified, apparently empty polyhedral shells of the *cbbL::Tc NC cbbL* and *cbbLS::Tc NC cbbLS* mutants were devoid of any detectible CbbL (Figure 5A,B) and CbbS polypeptides (Figure 5A). Likewise, immunoblots that were probed with the CbbM-specific antibody provided no evidence that Form II RubisCO was compartmentalized in carboxysomes (data not shown).

### CO_2_ fixation by mutant carboxysomes

To relate the observed growth rates of the mutants to the activity of the foreign and chimeric RubisCO species that are part of their carboxysomes, we quantified the CO_2_ fixation activities of intact mutant carboxysomes with a radiometric assay that measures the incorporation of radioactive bicarbonate into acid-stable products [6]. Not surprisingly, the empty carboxysome shells of the *cbbL::Tc NC cbbL*, *cbbLS::Tc NC cbbLS*, and *cbbLS::kan^r^* mutants did not have any detectable CO_2_ fixation activity (Table 1). The specific activity of carboxysomes purified from the *cbbS::Tc NC cbbS* mutant, which contained chimeric RubisCO, was only 10% of that exhibited by wild type carboxysomes. Likewise, carboxysomes obtained from the *cbbLS::Tc C cbbLS* mutant, which encapsulated heterologous *T. crunogena* carboxysomal RubisCO, were only approximately one fourth as active as their wild type counterparts. To correlate these values with the enzymatic activities of the respective purified RubisCO species, mutant carboxysomes were mechanically disrupted by a freeze-thaw treatment that releases the sequestered enzyme [24]. Shell remnants were removed by centrifugation, and the specific activities of the near-homogeneous RubisCO supernatant fractions determined as described [6]. RubisCO species freed from the broken carboxysomes of the *cbbS::Tc NC cbbS* and *cbbLS::Tc C cbbLS* mutants were catalytically compromised to a similar extent as they were in intact carboxysomes (Table 1).

**Table 1:** CO_2_ fixation activities of carboxysomes and RubisCO released from the microcompartments.

Mutant	Carboxysomes µmol min^−1^ mg^−1^	RubisCO µmol min^−1^ mg^−1^
*Wild type*	0.966±0.020	2.173±0.174
*cbbL::Tc NC cbbL*	0	- a)
*cbbS::Tc NC cbbS*	0.096±0.003	0.146±0.009
*cbbLS::Tc NC cbbLS*	0	- a)
*cbbLS::Tc C cbbLS*	0.234±0.003	0.295±0.010
*cbbL::kan^r^*	0	- a)

a) Only empty shells are formed in these mutants.

## Discussion

We have generated a battery of *H. neapolitanus* mutants in which one or both genes of the carboxysomal RubisCO were replaced with orthologs from *T. crunogena*. We show that the carboxysome of *H. neapolitanus* can accommodate chimeric and heterologous species of RubisCO and that it is the large subunit of these RubisCO species that determines whether the enzyme is sequestered into the microcompartments. The CO_2_ fixation activities of the mutant enzymes correlate with the growth rates of the mutants at ambient CO_2_ levels. Significantly, all mutants assemble stable carboxysome shells of apparently normal architecture and shape, demonstrating that α-carboxysome shell biogenesis and RubisCO sequestration are two independent processes.

### *H. neapolitanus* carboxysomes sequester foreign RubisCO

The *cso* operons of those autotrophic bacteria that form α-carboxysomes, the chemolithotrophs and some marine cyanobacteria (reviewed in [2]), contain *cbbL* and *cbbS* genes encoding the large and small subunit, respectively, of RubisCO. A notable exception is the *cso* operon of *Thiobacillus denitrificans*, which consists of a complete set of carboxysome shell genes but lacks those for the RubisCO subunits [15]. Interestingly, carboxysomes have not been detected to date in this bacterium [15]. In *H. neapolitanus*, all genes in the *cso* operon, including those that are present as multiple paralogs, are transcribed and therefore are likely important for microcompartment assembly, structure and/or function [20]. In light of these findings and considering the fact that the amino acid sequences of some carboxysome shell proteins are not particularly well conserved [15], it seems reasonable to assume that formation of carboxysomes relies on species-specific interactions of its protein constituents. Surprisingly, we found that the endogenous *H. neapolitanus* RubisCO can be replaced by the carboxysomal enzyme from *T. crunogena* in the carboxysomes of the *cbbLS::Tc C cbbLS* mutant. Aside from harboring the heterologous RubisCO, the mutant carboxysomes are very similar to their wild type counterparts with respect to shape, size and protein composition. The chimeric RubisCO composed of *H. neapolitanus* large subunit and the small subunit of the non-carboxysomal RubisCO from *T. crunogena* can likewise substitute for the endogenous enzyme in carboxysomes of the *cbbS::Tc NC cbbS* mutant.

The mutant carboxysomes that harbor heterologous and chimeric RubisCO species and their purified cargo enzymes are enzymatically active, but at reduced levels that correlate well with the slower growth rates of the respective mutants in air compared to wild type *H. neapolitanus*. The kinetic constants of the *T. crunogena* enzyme are yet to be determined, so one can only speculate about the reason for the poor performance of the enzyme in the *cbbLS::Tc C cbbLS* mutant. It is possible that the mild *hcr* phenotype of this mutant is a manifestation of a lower intrinsic carboxylation activity of the carboxysomal *T. crunogena* enzyme compared to that of its *H. neapolitanus* ortholog. Alternatively, the holoenzyme that assembles in the heterologous host may not be fully functional.

Low carboxylation activities have also been reported for RubisCO species that are composed of subunits from different origins [25]–[31]. For most of these chimeric enzymes, the molecular interactions between large and small subunits are less favorable than in the wild type protein and lead to holoenzymes of reduced stability [28]–[30]. The observed low specific activity of the mutant *H. neapolitanus* carboxysomes that contain the chimeric RubisCO was therefore not surprising. In fact, in electron micrographs of negatively stained carboxysomes purified from the *cbbS::Tc NC cbbS* mutant the characteristic donut-shaped RubisCO molecules that are clearly discernible in wild type microcompartments are not visible. Instead, their interiors appear to be filled with more irregularly shaped larger clusters that may represent protein aggregates with compromised enzymatic activity.

The failure of the *cbbLS::kan^r^* mutant to grow in ambient CO_2_ mirrors the growth behavior of the *hcr cbbL::*Km mutant reported by Baker *et al.* [10], which carries a kanamycin resistance cassette in the coding sequence of *cbbL* and does not produce carboxysomal RubisCO. Likewise, in the cyanobacterium *Synechocystis* 6803, replacement of the RubisCO *rbcL* gene, which encodes the large subunit of the Form I enzyme, by the *rbcM* gene for Form II RubisCO of the photosynthetic anaerobe *Rhodospirillum rubrum*, resulted in a “cyanorubrum” mutant that is not able to grow in air [21]. A similar “tobacco-rubrum” mutant also requires elevated CO_2_ levels for growth [32]. The severe *hcr* phenotypes of the mutants that cannot sequester their heterologous and chimeric RubisCO species into carboxysomes (*cbbL::Tc NC cbbL* and *cbbLS::Tc NC cbbLS*) may be related to the lower carboxylation efficiencies of noncarboxysomal RubisCO [14], [33], possibly exacerbated by holoenzyme assembly issues.

### CbbL determines whether RubisCO is incorporated into carboxysomes

The additional set of *cbbL* and *cbbS* paralogs in *T. crunogena* that is not part of the *cso* operon [19] was used to assess whether a noncarboxysomal RubisCO species can be incorporated into α-carboxysomes. In *H. marinus*, where the two Form I RubisCO gene sets are expressed under different environmental conditions [18], only the expression of the *cbbL* and *cbbS* copy in the *cso* operon correlates with carboxysome formation [18]. It is therefore generally assumed that only this RubisCO species is sequestered into the microcompartment. The single (non-*cso* associated) Form I RubisCO gene set of *T. denitrificans*, when expressed in a RubisCO null mutant of *Rhodobacter sphaeroides*, complements the mutant phenotype and was shown to yield active enzyme in the heterologous bacterium [33]. The subunits of this enzyme species apparently are able to assemble into functional holoenzymes in the foreign host, but since *R. sphaeroides* does not form carboxysomes this study did not address the question of enzyme sequestration. The results obtained with the *H. neapolitanus* RubisCO replacement mutants used in our study strongly imply that some structural feature unique to carboxysomal RubisCO protein and absent from the noncarboxysomal species is required for compartmentalization into carboxysomes.

Considering the high degree of primary structure conservation in CbbL polypeptides and the considerable sequence divergence in CbbS proteins (reviewed in [14]), one would predict that the small subunit determines whether a RubisCO species can be packaged into carboxysomes. A comprehensive comparison of large and small subunit amino acid sequences by Badger and Bek [14] revealed a six-amino acid insertion close to the N-terminus of noncarboxysomal CbbS proteins that is not present in carboxysomal small subunits. The authors suggest that this motif, which is predicted to be located on the surface of the folded polypeptide, might interfere with protein contacts necessary for encapsulation into carboxysome shells. Experimental evidence from our study shows that this is not the case for *H. neapolitanus* carboxysomes. *T. crunogena* noncarboxysomal CbbS, which contains these extra six amino acids (Supplemental Figure S1), is incorporated into carboxysomes in the *cbbS::Tc NC cbbS* mutant as part of a chimeric RubisCO holoenzyme featuring endogenous *H. neapolitanus* carboxysomal CbbL. Our finding suggests that the extra amino acids in noncarboxysomal CbbS do not interfere with holoenzyme sequestration into carboxysomes and that, instead, the large subunit of RubisCO determines if the holoenzyme is compartmentalized. Of the RubisCO replacement mutants we have studied, those that express endogenous or heterologous carboxysomal CbbL are able to incorporate the resulting RubisCO holoenzyme into their carboxysomes regardless of the associated CbbS species. By contrast, RubisCO sequestration does not occur in mutants in which the carboxysomal *cbbL* gene is replaced with a noncarboxysomal copy, even if it is paired with endogenous *H. neapolitanus* carboxysomal *cbbS*.

An alignment of the three carboxysomal and noncarboxysomal CbbL species used in this study (Supplemental Figure S1) does not reveal any striking differences that could explain why only the *T. crunogena* ortholog that is derived from its *cso* operon is packaged into *H. neapolitanus* carboxysomes. Because of this high degree of sequence conservation, any structural differences between carboxysomal and non-carboxysomal CbbL species that are important for compartmentalization are probably subtle. Their identification will therefore require a combination of approaches that include site-specific mutagenesis, elucidation of its crystal structure and a careful comparison with the known structure of the *H. neapolitanus* carboxysomal enzyme (PDB ID: 1SVD; Kerfeld, CA *et al*, 2005).

### Assembly of α-carboxysomal shells does not depend on the presence of RubisCO

The role of the carboxysomal RubisCO in microcompartment biogenesis and as a determinant of its architecture is not well understood. Price and Badger [34] observed circular structures resembling partially assembled carboxysome shell intermediates in thin sections of the filamentous cyanobacterium *Anabaena variabilis* M3 and proposed that the β-carboxysome shell is assembled prior to insertion of RubisCO molecules. The empty polyhedral structures that were observed in thin sections of *H. neapolitanus cbbLS::*Km mutant cells [10], and the arrangement of RubisCO holoenzymes in cryo-electron tomograms of carboxysomes [35] also favor a pathway of α-carboxysome formation that does not require a pre-assembled RubisCO core. Orús *et al.* [36], on the other hand, concluded that even the earliest β-carboxysome precursor stages visible in thin sections of *Synechococcus* PCC 7942 contain regular arrays of RubisCO and proposed that the shell forms around a RubisCO core. Recent *in vitro* pulldown experiments and *E. coli* co-expression studies of selected recombinant β-carboxysome components suggest that the large subunit of Form IB RubisCO can interact with other putative shell proteins, leading the authors to suggest a role for RbcL in β-carboxysome assembly [37], [38].

The *H. neapolitanus* RubisCO replacement mutants described in this study provide the first biochemical and ultrastructural characterization of purified polyhedral structures that are formed in the absence of any carboxysomal RubisCO protein and in the presence of compatible and incompatible RubisCO species. The empty shells found in the *cbbL::Tc NC cbbL*, *cbbLS::Tc NC cbbLS*, and *cbbLS::kan^r^* mutants are sufficiently stable to withstand the standard carboxysome purification procedure. Their existence clearly shows that α-carboxysome shell formation does not require a RubisCO assemblage and supports the biogenesis model proposed for β-carboxysomes by Price and Badger [34].

The possibility that the presence of RubisCO holoenzyme or of one of its subunits in amounts below our detection limit might serve as a trigger or scaffold for shell assembly was tested in the *cbbLS::kan^r^* mutant, which does not produce any Form I RubisCO protein. Clearly, the empty polyhedral shells formed in this mutant are assembled in the absence of cognate RubisCO holoenzyme and resemble those of wild type carboxysome shells in size, shape and protein composition. These results show that neither size nor polyhedral shape of the α-carboxysome shell depends on RubisCO molecules filling the interior microcompartment space. Similar observations were also made for the *pdu* microcompartment of *Salmonella enterica*. Deletion of the genes for the sequestered enzymes that participate in the catabolism of 1,2-propanediol do not affect the formation of shell structures [39].

Results from our study provide, for the first time, direct experimental evidence that α-carboxysome shell assembly and recruitment of RubisCO into the microcompartment interior are two independent processes. However, it must be emphasized that we do not advocate a sequential model for carboxysome biogenesis. Specific interactions of RubisCO with shell components are likely to guide sequestration of the enzyme into carboxysome shells *in vivo*. Contrary to sequence-based predictions that implicated the small subunit of the enzyme in determining whether a particular RubisCO species can be sequestered, we clearly identify the large subunit as the key to RubisCO incorporation into carboxysomes. Our discovery that the carboxysome of *H. neapolitanus* has the capacity to accommodate chimeric and heterologous RubisCO species opens the way to designing carboxysome-based microcompartments for nanotechnological applications such as custom chemical reactors or delivery vehicles.

## Materials and Methods

### Chemicals and reagents

Unless otherwise mentioned, chemicals and reagents were from Sigma, Fisher Scientific, Thermo Scientific (B-PER II and GelCode Blue stain, One-Step NBT-BCIP reagent, BCA protein assay reagents), Bio-Rad (Criterion PreCast 4–20% polyacrylamide gradient Tris-HCl protein gels), IDTDNA (oligonucleotides), New England Biolabs (restriction enzymes, DNA polymerase, DNA ligase), Agrisera (chicken anti-RbcL antibody, Product No. AS01 017), Cocalico Biologicals (rabbit anti-CsoS1 polyclonal antibody), Santa Cruz Biotechnology (goat anti-chicken and anti-rabbit HRP-tagged antibodies), Electron Microscopy Services (formvar coated copper grids, ammonium molybdate stain).

### Strains and growth conditions

All mutants described in this study were constructed with wild type *Halothiobacillus neapolitanus* (ATCC 23641). The culture medium and growth conditions were as described previously [6]. Growth of wild type and mutant cells was monitored by measuring the OD_600_.

### Construction of RubisCO mutants

Primers used to amplify the carboxysomal (C) and noncarboxysomal (NC) Form I RubisCO from *Thiomicrospira crunogena* XCL-2 (Tc) and the kanamycin resistance (*kan^r^*) cassette are listed in Table S1. Briefly, genes encoding the carboxysomal RubisCO (large subunit GenBank CP000109, GeneID: 3760532; small subunit GenBank CP000109, GeneID: 3760533) and noncarboxysomal RubisCO (large subunit GenBank CP000109, GeneID: 3761246; small subunit GenBank CP000109, GeneID: 3761247) were amplified from *Thiomicrospira crunogena* XCL-2 genomic DNA and cloned into the pCR-BluntII-TOPO vector (Invitrogen). The resulting insert was excised by digestion with BamHI and KpnI, and ligated along with a *kan^r^* cassette containing KpnI–XhoI ends into the BamHI–XhoI sites of the pPROEX-HTb vector (Invitrogen). The resulting construct was digested with BamHI and XhoI to release the insert. *Escherichia coli* DY330 cells [40] were co-transformed with this fragment and with pUC18 containing the *cbbL*-*cbbS* region of the *H. neapolitanus cso* operon to replace the wild type *cbbL*-*cbbS* region on the plasmid with that of the insert by homologous recombination. The resulting plasmid containing the mutated *cbbL-cbbS* region was electroporated into exponentially growing *H*. *neapolitanus* cells using the method of English *et al*. [12]. The presence of the desired changes in the *cso* operon of the *H. neapolitanus* mutants was confirmed by genomic sequencing (University of Maine DNA Sequencing Facility) and by PCR amplification.

### Protein analyses

Protein samples were resolved on 4–20% SDS-polyacrylamide gradient gels and stained with GelCode Blue. For detection of RubisCO large subunit, blots were probed with commercially available chicken polyclonal anti-RbcL IgY antibodies as primary antibody. Form II RubisCO (CbbM) and the CsoS1 shell proteins were detected using a commercial rabbit polyclonal anti-CbbM and polyclonal anti-CsoS1 antibodies generated in our laboratory, respectively. The secondary antibodies were tagged with horseradish peroxidase. Blots were developed using the One-Step NBT-BCIP reagent.

### Electron microscopy

Electron micrographs of wild type and mutant carboxysomes were taken as described previously [6] and scanned on an Epson Perfection V700 Photo flatbed scanner.

### CO_2_ fixation assays

Carboxysomes from wild type and mutant cells grown as 1.5 L batch cultures in air supplemented with 5% CO_2_ were isolated as described previously [24]. RubisCO species present in clarified cell extracts were separated by centrifugation at 27,000×g for 30 hours in 0.2 M–0.8 M sucrose/TEMB gradients (36 mL). The gradients were fractionated into 38 1-mL fractions. RubisCO activity in each fraction and in purified carboxysomes was assessed as described previously [6].

## Supporting Information

Table S1 (0.05 MB DOC)

Figure S1 (0.30 MB DOC)

## References

[1] Shively JM (2006). Complex Intracellular Structures in Prokaryotes..

[2] Heinhorst S, Cannon GC, Shively JM, Shively JM (2006). Carboxysomes and carboxysome-like inclusions.. Complex Intracellular Structures in Prokaryotes.

[3] Kerfeld CA, Sawaya MR, Tanaka S, Nguyen CV, Phillips M (2005). Protein structures forming the shell of primitive bacterial organelles.. Science.

[4] Tanaka S, Kerfeld CA, Sawaya MR, Cai F, Heinhorst S (2008). Atomic-level models of the bacterial carboxysome shell.. Science.

[5] Tsai Y, Sawaya MR, Cannon GC, Cai F, Williams EB (2007). The structure of the shell protein CsoS1A from *Halothiobacillus neapolitanus* and its implications for carboxysome function.. PLoS Biology.

[6] Dou Z, Heinhorst S, Williams EB, Murin CD, Shively JM (2008). CO_2_ fixation kinetics of *Halothiobacillus neapolitanus* mutant carboxysomes lacking carbonic anhydrase suggest the shell acts as a diffusional barrier for CO_2_.. J Biol Chem.

[7] Heinhorst S, Williams EB, Cai F, Murin CD, Shively JM (2006). Characterization of the carboxysomal carbonic anhydrase CsoSCA from *Halothiobacillus neapolitanus*.. J Bacteriol.

[8] Ludwig M, Sueltemeyer D, Price GD (2000). Isolation of *ccmKLMN* genes from the marine cyanobacterium, *Synechococcus* sp. PCC7002 (cyanophyceae), and evidence that CcmM is essential for carboxysome assembly.. Biochemistry.

[9] Badger MR, Hanson D, Price GD (2002). Evolution and diversity of CO_2_ concentrating mechanisms in cyanobacteria.. Funct Plant Biol.

[10] Baker SH, Jin S, Aldrich HC, Howard GT, Shively JM (1998). Insertion mutation of the form I *cbbL* gene encoding ribulose bisphosphate carboxylase/oxygenase (RuBisCO) in *Thiobacillus neapolitanus* results in expression of form II RuBisCO, loss of carboxysomes, and an increased CO_2_ requirement for growth.. J Bacteriol.

[11] Price GD, Howitt SM, Harrison K, Badger MR (1993). Analysis of a genomic DNA region from the cyanobacterium *Synechococcus* sp. strain PCC7942 involved in carboxysome assembly and function.. J Bacteriol.

[12] English RS, Jin S, Shively JM (1995). Use of electroporation to generate a *Thiobacillus neapolitanus* carboxysome mutant.. Appl Environ Microbiol.

[13] Andersson I, Backlund A (2008). Structure and function of Rubisco.. Plant Physiology and Biochemistry.

[14] Badger MR, Bek EJ (2008). Multiple Rubisco forms in proteobacteria: their functional significance in relation to CO_2_ acquisition by the CBB cycle.. J Exp Bot.

[15] Cannon GC, Baker SH, Soyer F, Johnson DR, Bradburne CE (2003). Organization of carboxysome genes in the thiobacilli.. Curr Microbiol.

[16] Cannon GC, Heinhorst S, Bradburne CE, Shively JM (2002). Carboxysome genomics: a status report.. Funct Plant Biol.

[17] Heinhorst S, Baker SH, Johnson DR, Davies PS, Cannon GC (2002). Two Copies of Form I RuBisCO Genes in *Acidithiobacillus ferrooxidans* ATCC 23270.. Curr Microbiol.

[18] Yoshizawa Y, Toyoda K, Arai H, Ishii M, Igarashi Y (2004). CO_2_-responsive expression and gene organization of three ribulose-1,5-bisphosphate carboxylase/oxygenase enzymes and carboxysomes in *Hydrogenovibrio marinus* strain MH-110.. J Bacteriol.

[19] Scott KM, Sievert SM, Abril FN, Ball LA, Barrett CJ (2006). The genome of deep-sea vent chemolithoautotroph *Thiomicrospira crunogena* XCL-2.. PLoS Biology.

[20] Cai F, Heinhorst S, Shively J, Cannon G (2008). Transcript analysis of the *Halothiobacillus neapolitanus cso* operon.. Arch Microbiol.

[21] Pierce J, Carlson TJ, Williams JGK (1989). A cyanobacterial mutant requiring the expression of ribulose bisphosphate carboxylase from a photosynthetic anaerobe.. Proc Natl Acad Sci U S A.

[22] Amichay D, Levitz R, Gurevitz M (1993). Construction of a *Synechocystis* PCC6803 mutant suitable for the study of variant hexadecameric ribulose bisphosphate carboxylase/oxygenase enzymes.. Plant Mol Biol.

[23] Cannon GC, Shively JM (1983). Characterization of a homogeneous preparation of carboxysomes from *Thiobacillus neapolitanus*.. Arch Microbiol.

[24] So AK, Espie GS, Williams EB, Shively JM, Heinhorst S (2004). A novel evolutionary lineage of carbonic anhydrase (epsilon class) is a component of the carboxysome shell.. J Bacteriol.

[25] Andrews TJ, Lorimer GH (1985). Catalytic properties of a hybrid between cyanobacterial large subunits and higher plant small subunits of ribulose bisphosphate carboxylase-oxygenase.. J Biol Chem.

[26] Kanevski I, Maliga P, Rhoades DF, Gutteridge S (1999). Plastome engineering of ribulose-1,5-bisphosphate carboxylase/oxygenase in tobacco to form a sunflower large subunit and tobacco small subunit hybrid.. Plant Physiol.

[27] Getzoff TP, Zhu G, Bohnert HJ, Jensen RG (1998). Chimeric *Arabidopsis thaliana* ribulose-1,5-bisphosphate carboxylase/oxygenase containing a pea small subunit protein is compromised in carbamylation.. Plant Physiol.

[28] Read BA, Tabita FR (1992). A hybrid ribulosebisphosphate carboxylase/oxygenase enzyme exhibiting a substantial increase in substrate specificity factor.. Biochemistry.

[29] van der Vies SM, Bradley D, Gatenby AA (1986). Assembly of cyanobacterial and higher plant ribulose bisphosphate carboxylase subunits into functional homologous and heterologous enzyme molecules in *Escherichia coli.*. Embo J.

[30] Wang Y-L, Zhou J-H, Wang Y-F, Bao J-S, Chen H-B (2001). Properties of hybrid enzymes between *Synechococcus* large subunits and higher plant small subunits of ribulose-1,5-bisphosphate carboxylase/oxygenase in *Escherichia coli*.. Arch Biochem Biophys.

[31] Karkehabadi S, Peddi SR, Anwaruzzaman M, Taylor TC, Cederlund A (2005). Chimeric small subunits influence catalysis without causing global conformational changes in the crystal structure of ribulose-1,5-bisphosphate carboxylase/oxygenase.. Biochemistry.

[32] Whitney SM, Andrews TJ (2003). Photosynthesis and growth of tobacco with a substituted bacterial Rubisco mirror the properties of the introduced enzyme.. Plant Physiol.

[33] Hernandez JM, Baker SH, Lorbach SC, Shively JM, Tabita FR (1996). Deduced amino acid sequence, functional expression, and unique enzymatic properties of the form I and form II ribulose bisphosphate carboxylase/oxygenase from the chemoautotrophic bacterium *Thiobacillus denitrificans*.. J Bacteriol.

[34] Price GD, Badger MR (1991). Evidence for the role of carboxysomes in the cyanobacterial CO_2_-concentrating mechanism.. Can J Bot.

[35] Iancu CV, Ding HJ, Morris DM, Dias DP, Gonzales AD (2007). The structure of isolated *Synechococcus* strain WH8102 carboxysomes as revealed by electron cryotomography.. J Mol Biol.

[36] Orus MI, Rodriguez ML, Martinez F, Marco E (1995). Biogenesis and ultrastructure of carboxysomes from wild type and mutants of *Synechococcus* sp. strain PCC 7942.. Plant Physiol.

[37] Long BM, Badger MR, Whitney SM, Price GD (2007). Analysis of Carboxysomes from *Synechococcus* PCC7942 Reveals Multiple Rubisco Complexes with Carboxysomal Proteins CcmM and CcaA.. J Biol Chem.

[38] Cot SS-W, So AK-C, Espie GS (2008). A Multiprotein Bicarbonate Dehydration Complex Essential to Carboxysome Function in Cyanobacteria.. J Bacteriol.

[39] Bobik TA (2006). Polyhedral organelles compartmenting bacterial metabolic processes.. Appl Microbiol Biotechnol.

[40] Yu D, Ellis HM, Lee E-C, Jenkins NA, Copeland NG (2000). An efficient recombination system for chromosome engineering in *Escherichia coli*.. Proc Natl Acad Sci U S A.

