# Supplementary material for: Halothiobacillus neapolitanus Carboxysomes Sequester Heterologous and Chimeric RubisCO Species
Source: PLoS One. 2008 Oct 30;3(10):e3570. doi: 10.1371/journal.pone.0003570 (PMC2570492; doi:10.1371/journal.pone.0003570)
Supplement: Table S1 — (0.05 MB DOC) [file pone.0003570.s001.doc]

**Supplementary Table S1. Oligonucleotides used for constructing *Halothiobacillus neapolitanus*** FI RubisCO mutants.

| **Target gene** | **Primer name** | **Primer sequence** | |
| --- | --- | --- | --- |
| ***cbbL::Tc NC cbbL*** | | | |
| *T. crunogena NC cbbL* | *NC cbbL F* | *GGATCC*CACAGGGGCGTTGCGCGAATCCCCCATCCTTTCAGGAGGAACTCATGGCTAAGACTTATAACGCCGGTG | |
|  | *NC cbbL R* | *GGTACC*TTACTTATGCTTAACATCTAGCTTGTCAACTGTGTCGAATTCGAACTTGATTTCTTTCCAAGTTTCCAT | |
| *Kanamycin gene* | *NC cbbL-kan^r^ F* | *GGTACC*CCGGAATTGCCAGCTGGGGCGCCCTCTGGTAAGGTTGGGAAGCCCTGCAAAGTAAACTGGATGGCTTTC | |
|  | *NC cbbL-kan^r^ R* | *CTCGAG*GCATTTCAGCCATGGTTACTCACCTTAGTATGTTGTGTGGTACGAGGGATCAGAAGAACTCGTCAAGAA | |
| ***cbbS::Tc NC cbbS*** | | | |
| *T. crunogena NC cbbS* | *NC cbbS F* | *GGATCC*CGTTGATCCCTCGTACCACACAACATACTAAGGTGAGTAACCATGAGTATTCAAGATTACCCATCTC | |
|  | *NC cbbS R* | *GGTACC*TTACATGTCACCACGCTTAACAAGCATGTTAGCACCTTGAGACTGTGCATAGTTATCATAACCAATCAAAC | |
| *Kanamycin gene* | *NC cbbS-kan^r^ F* | *GGTACC*CCGGAATTGCCAGCTGGGGCGCCCTCTGGTAAGGTTGGGAAGCCCTGCAAAGTAAACTGGATGGCTTTC | |
|  | *NC cbbS-kan^r^ R* | *CTCGAG*TGAATAAGTGCTGTGCGTAGAGAAACGCACAGCGCAATGACAGACTTGACTCAGAAGAACTCGTCAAGAA | |
| ***cbbLS::Tc NC cbbLS*** | | | |
| *T. crunogena NC cbbLS* | *NC cbbLS F* | *GGATCC*CACAGGGGCGTTGCGCGAATCCCCCATCCTTTCAGGAGGAACTCATGGCTAAGACTTATAACGCCGGTG | |
|  | *NC cbbLS R* | *GGTACC*TTACATGTCACCACGCTTAACAAGCATGTTAGCACCTTGAGACTGTGCATAGTTATCATAACCAATCAAAC | |
| *Kanamycin gene* | *NC cbbLS-kan^r^ F* | *GGTACC*CCGGAATTGCCAGCTGGGGCGCCCTCTGGTAAGGTTGGGAAGCCCTGCAAAGTAAACTGGATGGCTTTC | |
|  | *NC cbbLS-kan^r^ R* | *CTCGAG*TGAATAAGTGCTGTGCGTAGAGAAACGCACAGCGCAATGACAGACTTGACTCAGAAGAACTCGTCAAGAA | |
| ***cbbLS::Tc C cbbLS*** | | | |
| *T. crunogena C cbbLS* | *C cbbLS F* | *GGATCC*CGTTGATCCCTCGTACCACACAACATACTAAGGTGAGTAACCATGGCAAGTAAAACGTTTGATGCTG | |
|  | *C cbbLS R* | *GGTACC*TTACATGCCACGTGGGCGATAAACCACAAAGTTGTGACCTTGGCACTGAGTATAGTTGTCATACCCAACCA | |
| *Kanamycin gene* | *C cbbLS-kan^r^ F* | *GGTACC*CCGGAATTGCCAGCTGGGGCGCCCTCTGGTAAGGTTGGGAAGCCCTGCAAAGTAAACTGGATGGCTTTC | |
|  | *C cbbLS-kan^r^ R* | *CTCGAG*TGAATAAGTGCTGTGCGTAGAGAAACGCACAGCGCAATGACAGACTTGACTCAGAAGAACTCGTCAAGAA | |
| ***cbbLS::kan^r^*** | | | |
| *Kanamycin gene* | *kan^r^ F* | | *GGATCC*CAAGCCACAGGGGCGTTGCGCGAATCCCCCATCCTTTCAGGAGGAACTCCCGGAATTGCCAGCTGGGGC |
|  | *kan^r^ R* | | *CTCGAG*TGAATAAGTGCTGTGCGTAGAGAAACGCACAGCGCAATGACAGACTTGACTCAGAAGAACTCGTCAAGAA |

Underlined sequences bear homology to the 5’ and 3’ flanking regions of the *cbbL* and *cbbS* genes in *Halothiobacillus neapolitanus*.

Italicized sequences represent the BamHI, KpnI, and XhoI restriction sites used for cloning purposes.
