# Supplementary material for: Halothiobacillus neapolitanus Carboxysomes Sequester Heterologous and Chimeric RubisCO Species
Source: PLoS One. 2008 Oct 30;3(10):e3570. doi: 10.1371/journal.pone.0003570 (PMC2570492; doi:10.1371/journal.pone.0003570)
Supplement: Figure S1 — (0.30 MB DOC) [file pone.0003570.s002.doc]

**Supplementary Figure 1**

**
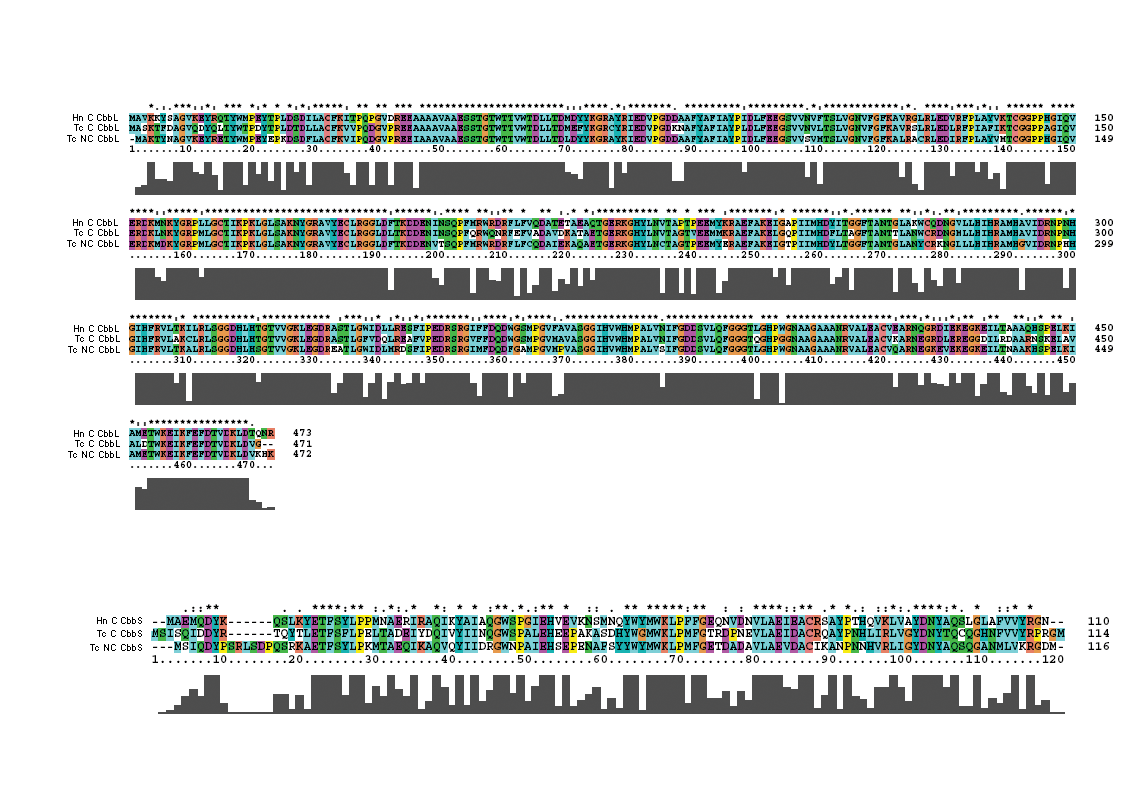
**

**Supplemental Figure S1. Alignment of FI RubisCO large and small subunits**

Amino acid sequences of carboxysomal (C) and noncarboxysomal (NC) large (CbbL) and small (CbbS) RubisCO subunits from *H. neapolitanus* (Hn) and *T. crunogena* (Tc) were aligned using ClustalX 1.83.
